# The effect of ENSO on common bean production in Colombia: a time series approach

**DOI:** 10.1007/s12571-022-01290-z

**Published:** 2022-06-10

**Authors:** Hernan Botero, Andrew P. Barnes

**Affiliations:** 1 Environment and Society Research Group, Kings Buildings Campus, Research Associate in the Rural Economy, Environment and Society (REES) Group, Scotland’s Rural College (SRUC), West Mains Road, Edinburgh, UK; 2 Head of Department of Rural Economy, Environment and Society (REES) Group, Scotland’s Rural College (SRUC), SRUC, Kings Buildings Campus, West Mains Road, EH9 3JG Edinburgh, UK

**Keywords:** Environment and Growth, Latin America, ENSO, Land Use Patterns, Common Beans, O44, O54, Q54, R14, O13

## Abstract

The common bean is an important staple food in Colombia with diverse nutritional content and environmental benefits. The most important climatic risk confronted by common bean production in Colombia is El Niño Southern Oscillation (ENSO) since its two extreme phases —El Niño and La Niña— increase the intensity and variety of abiotic and biotic stresses in the region. Using information from the Food and Agricultural Organization (FAO) for the period 1991–2018, we test whether pre-2030 ENSO has had a negative impact on common bean production in Colombia using a Prais–Winsten regression model. We find that common beans’ yields have been negatively affected by El Niño, but not by La Niña. Moreover, short-run ENSO-induced deviations in the growth rate of precipitation with respect to its long-run value reduce yields and increase farmers’ income from common bean production. These results have two important implications. From a modelling standpoint, we find that precipitation has a non-linear relationship with yields and incomes, implying that second-order effects should be incorporated in any analysis of the effects of climatic variables on agricultural production. From a policy perspective, our results suggest a need for countercyclical polices to counteract price spikes of common beans in the Colombian market since, when they occur, they tend to over-compensate the reduction in yields, which reduce common bean consumers’ purchasing power and food security.

## Introduction

The common bean (Phaseolus vulgaris L.) is an important cash crop and a key source of nutrition in developing countries across the Global South (Porch et al., 2013; Myers & Kmiecik, 2017). Its popularity arises from the fact that it is very nutritious and relatively easy to grow. The common bean plant produces immature pods that contain protein, carbohydrates, vitamin C and K, and carotenoids, and dry beans that contain protein, several micronutrients —such as calcium, iron, magnesium, potassium, and phosphorus—, carbohydrates, and soluble fiber (USDA, 2015). In addition, as the common bean plant has a natural ability to fixate nitrogen to the soil, it can easily be incorporated in cropping systems to reduce dependence on inorganic nitrogen and its concomitant environmental problems (Briggs et al., 2005; Williams et al., 2017).

Extreme climatic events threaten the use of common beans as a staple crop with its positive environmental externalities in Colombia (Camargo & Alonso, 2009; Ianneta et al*.*, 2013). The El Niño-Southern Oscillation (ENSO) is a key driver behind interannual climate variability in the southern Pacific Ocean, affecting the climatic conditions in South America through teleconnections between geographically separated regions (Zebiak et al*.*, 2015; Merchant et al*.*, 2019). Hence, ENSO is the most important climatic risk confronted by Colombian common bean growers (Bruinsma, 2003, Chapter 13; Ramirez-Villegas et al*.*, 2012; Eitzinger et al*.*, 2014; Feola et al., 2015; Ramirez-Cabral et al*.*, 2016; Güiza-Villa et al., 2020). It is characterized by two extreme phases and a neutral one (Song & Son, 2018). The two extreme phases are known as El Niño (the warm/dry phase) and La Niña (the cool/wet phase) and occur irregularly every three to seven years, with El Niño being the most prevalent phase of the two (Ropelewski & Halpert, 1987; Aceituno, 1988; Camilloni & Barros, 2000; Grimm & Tedeschi, 2009).

ENSO generates two differing climatic conditions in Colombia (Smith & Ubilava, 2017, Figure A1, page 160). During El Niño, trade winds weaken in the tropical Pacific Ocean, generating a generalised increase in temperatures and a generalised reduction in cumulative precipitation in the entire Colombian territory, making droughts more prevalent. During La Niña, trade winds intensify in the tropical Pacific Ocean, producing a generalised reduction in temperatures and a generalised increase in cumulative precipitation, which is positively correlated with more events of floods and landslides in the entire Colombian territory. Consequently, ENSO may affect common bean production in Colombia through several channels. During El Niño, drier weather conditions may potentially affect vegetative grow, pod formation, and pod filling (Rosenzweig et al*.*, 2001; Beebe et al*.*, 2011; Castro-Guerrero et al*.*, 2016). During La Niña, wetter weather conditions may generate an increased prevalence of plant pests and diseases (Rosenzweig et al*.*, 2001; Ramirez-Villegas et al*.*, 2012; Feola et al*.*, 2015). As a result, both extreme phases of ENSO can reduce Colombian common bean production through an increased prevalence of abiotic and biotic stresses that affect crop health and yields. In addition, ENSO-induced yield reductions can lead to a spike in the prices of common beans, which reduce consumers’ purchasing power and impact the terms of trade (Brunner, 2002; Ubilava & Holt, 2013; Cashin et al*.*, 2017; Ubilava, 2017). Both price inflation and terms of trade are two factors that in turn may influence farmers’ income from common bean production through an increased competition of international varieties in the Colombian market (Barro, 1996; Bruinsma, 2003, chapters 9 and 10).

**Fig. 1:**
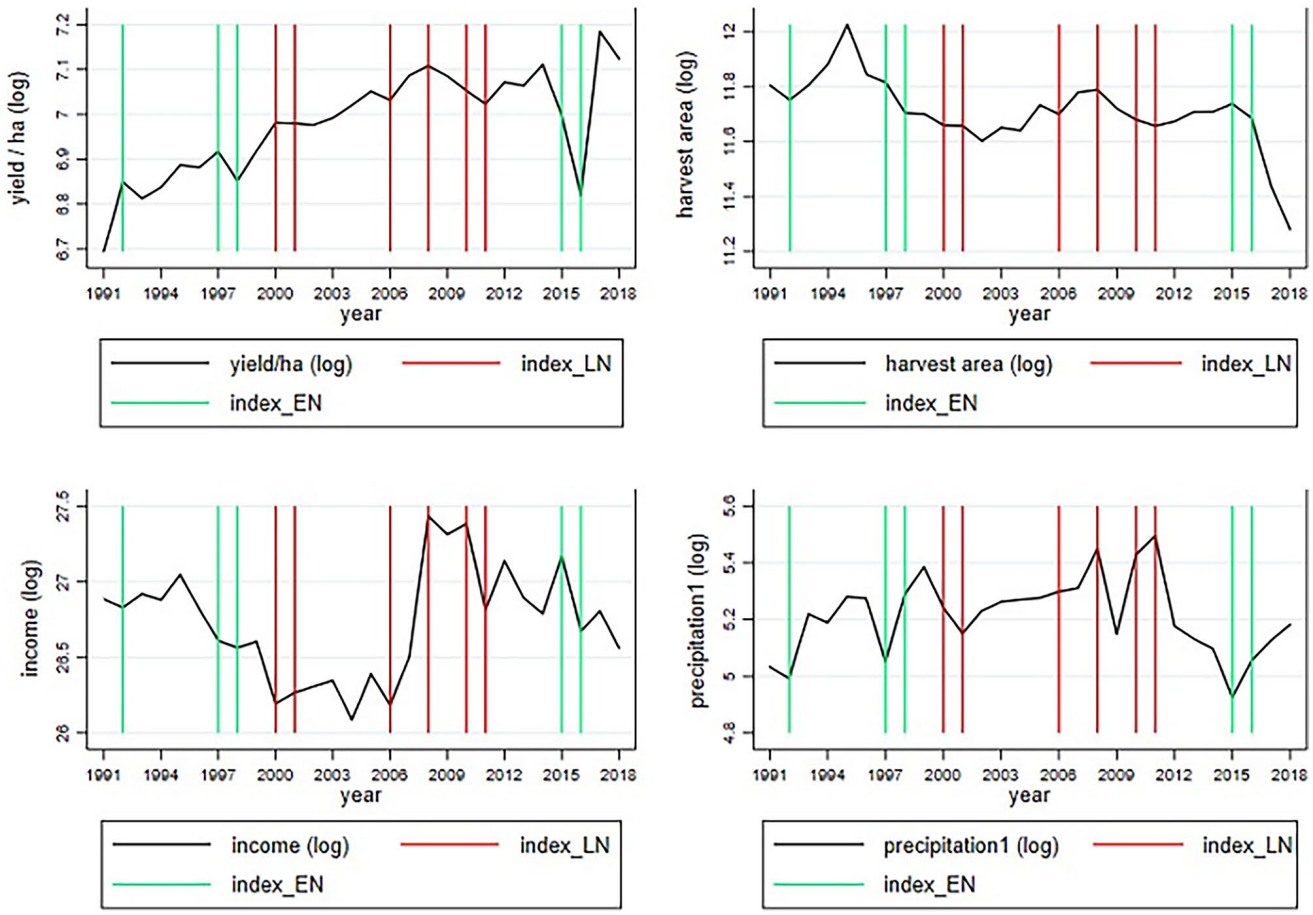
Common beans’ yield/ha, harvest area, income, precipitation, and ENSO indices in Colombia for the period 1991–2018

FAO forecasted in 2003 that common beans’ yields were expected to be negatively affected during ENSO’s extreme phases prior to 2030, but that farmers’ incomes from common bean production were not going to be affected by these phases (Bruinsma, 2003). The argument was that price spikes would just compensate yields’ reductions, leaving farmers’ incomes unaffected. These price spikes were also expected to reduce common bean consumers’ purchasing power and food security. No study has attempted to test this prediction yet. However, risk assessments of ENSO-related climatic variations on common bean production are necessary for at least three reasons: a) they help to understand how ENSO-related climatic anomalies reduce common bean yields and increase the prices of common beans, which reduce food security in a region where common beans represent an important source of plant-based protein (Porch et al., 2013; Myers & Kmiecik, 2017); b) they help to understand how farmers’ incomes from common bean production are affected, which in turn influences the design of counter-cyclical economic policy aimed at reducing the negative effects of climatic events on farmers’ income streams; and, c) they inform policy design and adaptation and mitigation strategy selection aimed at ameliorating the short- and long-term negative consequences of ENSO-related shocks on common bean availability and affordability by low- and middle income households (Gupta et al*.*, 2014; Garcia-Romero & Molina, 2015). In order to perform these assessments, a quantitative evaluation of ENSO-related effects on common bean production is required.

This study performs the first quantitative evaluation of the effects of ENSO on common bean production in Colombia. It aims to identify the impact of pre-2030 ENSO-related climatic anomalies on common beans’ yields and prices and on the incomes received by common bean farmers in Colombia. This analysis is a key element for the design of current and future public policies conducive to ameliorate the negative effects of ENSO-related climatic anomalies on food security in the country since current ENSO-related effects are expected to be magnified with more intense ENSO events associated with a worsened climate change. To accomplish it, this paper performs the first econometric estimation of the effect of ENSO on common bean production in Colombia, employing a times series approach and information for the period 1991–2018. To capture the occurrence of ENSO, two measures are computed: a) an index that captures the occurrence of ENSO, which is based on the distribution of the Southern Oscillation Index (SOI) (Restrepo et al*.*, 2014); and b) a cumulative measure of precipitation, which is used as a proxy for the effect of ENSO on rainfall distribution in Colombia. As there is only information on national averages for the variables of interests in FAO database, a Prais–Winsten regression model is employed in order to control for an omitted variable bias problem generated by a lack of regressors that capture input use at farm level (Greene, 2003, p. 148).

## Conceptual framework

ENSO significantly affects Colombian precipitation patterns and hydrological cycles, greatly impacting its agricultural production and food prices (Restrepo & Kjerfve, 2000; Tol, 2009; Melo-León et al*.*, 2017; Bedoya-Soto et al*.*, 2019). There have been several attempts to unveil the causal relationship between ENSO and agricultural production in Colombia, with most studies focusing only on total agricultural GDP (MinAgricultura, 2012; Melo-León et al*.*, 2017; Smith and Ubilava, 2017) or food inflation (Abril-Salcedo et al*.*, 2018). Some studies have also been performed to analyse the effect of ENSO on particular agricultural products, such as coffee (Bastianin et al*.*, 2018), citrus (Gonzalez-Orozco et al*.*, 2020), oil palm tree (Cadena et al*.*, 2006), avocado (Rodriguez-Gil et al*.*, 2020), and tilapia (Blanco et al*.*, 2007). To our knowledge, there has been no attempt to analyse the effect of ENSO on common bean production using a time series approach. The closest attempt to perform an analysis of the effect of ENSO on common bean production is performed by Esquivel et al*.* (2018) who assess seasonal precipitation predictability for key growing areas of rice, maize, and common beans. In this study, the authors compare the actual precipitation distribution with the predicted one during ENSO in order to determine how accurate yield predictions that use modelled precipitation are. Another close attempt is Ramirez-Villegas et al*.* (2012) who estimate the effect of climate change on Colombian agricultural production areas by 2050. In this study, the authors investigate the effect of ENSO on precipitation, temperature, and productivity of key agricultural products, such as maize and common beans. These authors forecast the yields of common beans for 2050 given IPCC’s estimations of future relationships between climate change, ENSO dynamics, and precipitation dynamics in the region. IDEAM (2013) also assesses the risks imposed by climate change on common bean production in Colombia, estimating that water deficits and excesses generated by ENSO may have negative consequences on future yields. None of these studies has attempted to use a time series approach to determine the effect of ENSO on common bean production.

Evidence for Latin America indicates that ENSO and climate change are expected to reduce common bean production in Colombia. Using two different global climate models, Ramirez-Cabral et al*.* (2016) forecast that common bean production will be mainly concentrated in South-Western Colombia near the frontier with Ecuador, disappearing almost entirely from Central and Northern Colombia by 2100. According to these authors, ENSO will be the major climatic risk confronted by common bean production in the region, becoming the main driver behind crop area displacement after 2050. In turn, Cirino et al*.* (2015) assess the impact of ENSO-related climate variations on agricultural production in Brazil. The authors find that common bean production is very vulnerable to El Niño effects in the Northeast region, generating productivity losses of up to 50% due to droughts and floods. As Northeastern Brazil has similar weather patterns than Northern Colombia, these results are expected to apply to common bean production in the whole geographical area, not only to the Brazilian portion of the territory. In addition, Ruiz and Pabon (2013) show that common bean production is highly affected by water excesses and deficits produced by the two extreme phases of ENSO in the Colombian department of Atlántico. Thus, both El Niño and La Niña phases seem to have a negative impact on common bean production in Northern Colombia. Finally, Lizumi et al*.* (2014) estimate that ENSO will have a significant negative impact on most growing areas in Colombia after 2050, reducing the yields of the most important crops such as maize, rice, wheat, and soybeans. These authors also show that both extreme phases of ENSO will have a negative impact on soybeans, inducing a reduction in the area under these crops in Colombia.

In the absence of previous work that apply the regression approach to analyse the effect of ENSO on common bean production, we are forced to rely on the general literature to conceptualise the problem at hand. Several approaches to analyse the effect of ENSO on agricultural production using regression analysis have been proposed in the literature. Dell et al*.* (2014) summarise them. All proposed approaches proceed in a similar fashion: They select a relevant endogenous variable (GDP, inflation, etc.), build an index or rely on a proxy of ENSO – such as precipitation levels – to capture the occurrence of ENSO, and introduce additional regressors that control for other phenomena that also help explain the variability of the endogenous variable selected for analysis. They differ in the statistical methodology employed to control for serial autocorrelation and heteroscedasticity, and in the use of cross-sectional or longitudinal data, time series, or panel data to run the estimations. They also differ in whether the variables used are expressed in levels or in differences, or in the type of indices used to capture the occurrence of ENSO. All these details must be considered in order to produce unbiased and consistent estimates of the effect of ENSO on common bean production. We select a time series approach because there is only information for common bean production for the period 1991–2018 at national level, which represents the average values for the Colombian common bean regional markets. Section 3 explains the variables selected and Section 4 explains in more detail the statistical model utilised.

Regardless of the methodology employed, our main focus in this study is Colombian consumers’ food security and our secondary focus is Colombian farmers’ income streams from common bean production. Following the discussion proposed by Bruinsma (2003), we focus attention on common beans’ yields because they determine food availability. Lower yields are expected to reduce availability, which reduces consumers’ access to food (Eitzinger et al*.*, 2014). We also focus attention on farmers’ incomes from common bean production for two reasons. On the one hand, farmers’ incomes directly reflect the economic impact of ENSO on farmers’ livelihoods (Feola et al*.*, 2015). If ENSO-related weather anomalies reduce farmers’ incomes from common bean production, ENSO phases also have a negative impact on farmers’ livelihoods. On the other hand, farmers’ incomes indirectly capture the affordability of common beans (Aronson, 2020). If farmers receive a larger income from common bean production during an ENSO-induced negative weather shock that simultaneously reduce yields and increase prices, they are transferring the reduction on yields to consumers, which negatively impacts the affordability of the commodity.

## Data

Two endogenous variables have been selected for this study: yield/ha and farmers’ income from common bean production. The selection of these variables responds to the discussion contained in the previous section. Information on common beans’ yield/ha for Colombia is obtained from FAO’s dataset, which contains information on the annual average number of kg of common beans obtained per hectare for the period 1991 to 2018. This dataset also contains information on the annual number of hectares of land harvested with common beans and the annual average price per kg of common beans for the same period. All these values are used to compute the total annual income that Colombian farmers obtain from common bean production. Table 1 contains the formula used to compute farmers’ income and the definition of the endogenous variables used in this study.

**Table 1:** Definition of the variables used in this study (annual values for the period 1991–2018)

Names	Units	Definitions
yield/ha	kg/year	Yield of Common Beans per hectare expressed in kilograms per year
harvest area	ha/year	Total number of hectares under common beans per year
income	(LCU^b^; 2016 = 100)	Farmers’ income from common bean productions per year expressed in Colombian pesos of 2016 [= (harvest area)*(yield/ha)*(price/kg)]
phosphorus/ha	kg/ha	Kg of phosphorus per hectare per year
fungicide/ha	lt/ha	Lt of fungicide per hectare per year
pesticide/ha	kg/ha	Kg of pesticide per hectare per year
gov. consumption	(%; 2016 = 100)	Total government consumption as a percentage of the total GDP^a^
workers/ha	ratio	Number of workers per hectare per year
private credit	(%; 2016 = 100)	Domestic credit provided by the financial sector as a percentage of GDP^a^
agricultural land	%	Percentage of total land dedicated to agricultural production
precipitation1	annual mm	total annual precipitation (all provinces; all year)
precipitation2	annual mm	total annual precipitation (13 most important provinces for common bean production; all year)
precipitation3	annual mm	total annual precipitation (13 most important provinces for common bean production; growing and flowering periods: April, May, June, July, August, and September)
index_La Niña	dichotomous	assumes the value of 1 if La Niña occurs in a particular year, and 0 otherwise
index_EL Niño	dichotomous	assumes the value of 1 if El Niño occurs in a particular year, and 0 otherwise
index_ENSO	dichotomous	assumes the value of 1 if either El Niño or La Niña occur in a particular year, and 0 otherwise

^a^ Gross Domestic Product

^b^ *LCU* Local Currency Units

Table 2 presents descriptive statistics of all the variables used in this study. The table shows that the distribution of the growth rate of yield/ha for the period 1991–2018 is symmetric around its average value of 2%. In addition, the growth rate of yield was smaller than $-3\%$ in 25% of the years considered in the sample and larger than 5% in 25% of the years considered. This distribution confirms FAO’s forecast that common beans’ yields were going to growth constantly prior to 2030, exhibiting a growth rate with a stable variation around a positive long-run mean. In contrast, FAO’s forecast fails to account for movements in farmers’ income from common bean production. This variable grew at an average of -1.2% in the period 1991–2018, exhibiting a very volatile distribution. This average value shows that Colombian farmers received a reduced income from common bean production in the period under analysis. It remains to determine whether these changes are driven by ENSO-related climatic variations, which is performed in the next section.

**Table 2:** Descriptive Statistics of the variables used

Variables^a^	Mean		Std		Min		1st Quartile		Median		3er Quartile		Max	
	Levels	Differences	Levels	Differences	Levels	Differences	Levels	Differences	Levels	Differences	Levels	Differences	Levels	Differences
yield/ha	6.98	0.02	0.12	0.10	6.69	-0.18	6.89	-0.03	7.00	0.02	7.07	0.05	7.19	0.37
harvest area	11.71	-0.019	0.13	0.085	11.28	-0.245	11.66	-0.052	11.71	-0.011	11.78	0.035	12.03	0.143
income	26.73	-0.012	0.37	0.309	26.09	-0.569	26.45	-0.227	26.80	-0.040	26.91	0.133	27.43	0.934
phosphorus/ha	4.01	0.047	1.61	0.992	2.13	-2.83	2.71	-0.340	3.58	0.080	4.68	0.300	7.17	2.440
fungicide/ha	0.45	0.000	0.29	0.257	0.14	-0.710	0.16	-0.100	0.47	0.000	0.64	0.150	1.05	0.580
pesticide/ha	1.24	0.014	0.73	0.363	0.33	-0.900	0.59	-0.050	1.18	0.040	1.82	0.150	2.77	0.970
gov. consumption^b^	2.89	0.007	0.19	0.115	2.56	-0.154	2.74	-0.091	2.89	0.000	3.04	0.074	3.18	0.336
workers/ha	0.09	0.001	0.01	0.005	0.07	-0.010	0.09	0.000	0.09	0.000	0.09	0.000	0.1	0.010
private credit^b^	3.45	0.028	0.27	0.106	3.04	-0.413	3.19	-0.003	3.45	0.048	3.61	0.078	3.91	0.183
agricultural land^b^	3.68	0.004	0.05	0.027	3.62	-0.072	3.64	-0.002	3.70	0.000	3.70	0.008	3.80	0.101
precipitation1	11.21	0.005	0.13	0.141	10.94	-0.306	11.13	-0.042	11.22	0.013	11.28	0.083	11.48	0.267
precipitation2	10.25	0.004	0.16	0.176	9.92	-0.358	10.17	-0.125	10.26	0.017	10.32	0.109	10.58	0.296
precipitation3	9.60	0.006	0.19	0.243	9.22	-0.595	9.47	-0.145	9.61	-0.015	9.72	0.237	10.05	0.454
Indices^c^	Categories													
	0	1												
index_La Niña	23	5												
index_EL Niño	22	6												
index_ENSO	17	11												

^a^ All variables are introduced in logarithms

^b^ Variables expressed in percentages were multiplied by 100 before taking logarithms

^c^ Indices are introduced in levels as defined in Table 1

In turn, Dell et al*.* (2014) propose several alternatives to capture the occurrence of ENSO. The simplest one involves generating a dichotomous variable that captures both the occurrence of La Niña and El Niño phases, without differentiating between them. In order to determine the occurrence of ENSO, we rely on the monthly distribution of the Southern Oscillation Index (SOI) reported by the US National Oceanic and Atmospheric Administration (NOAA, 2021). We initially determine the occurrence of ENSO at monthly level by placing a 1 in those months in which the value of SOI is larger or smaller than $\mu \pm 2*\sigma$ —where $\mu$ and $\sigma$ represent the mean and standard deviation of the monthly SOI for the period 1991 to 2018—, and 0 otherwise. Then, the annual index is constructed by placing a 1 in all those years in which ENSO occurred at least once during the course of the year, as captured by the monthly index previously constructed.

One important drawback of the latter dichotomous variable is that it cannot capture the effect of La Niña and El Niño phases separately. Hence, if this variable is statistically significant, it only captures ENSO incidence on common bean production or income, without specifying which phase is the one affecting production or income. Another alternative is to define a dummy variable for each extreme phase of ENSO. The annual indices are constructed by placing a 1 in all those years in which El Niño (La Niña) occurred at least once during the course of the year —as captured by the monthly index—, and 0 otherwise. Defined in this way, these variables capture how much production/income deviates from the other states when El Niño (La Niña) occurs. Figure 2 depicts both the El Niño and La Niña indices that result from the previous computations. It is worth noting that, in some instances, both extreme phases seem to last two years. This result can be explained by the fact that some phases lasted more than 10 months, passing from one year to the next one, affecting the value that the annual index in both consecutive years assumes. We decided to maintain the ones in both consecutive years because ENSO has been found to have up to a six-month-lagged effect on precipitation in the continent (Abril-Salcedo et al*.*, 2018). Computing ENSO events in this way captures five events of La Niña and six events of El Niño in the period 1991–2018, which implies that El Niño has been the more prevalent event in the period under analysis. In addition, there has been an extreme ENSO event in 11 years out of the 28 years considered in the sample, which implies that ENSO has a large incidence on Colombian agricultural production and has become more recurrent in the last 30 years.

One drawback of utilising dummy variables to capture the occurrence of ENSO is that they do not inform whether the change in the endogenous variable induced by ENSO is given by excessive or insufficient rainfall. A possible solution is to introduce variables that proxy for climatic phenomena produced by ENSO. According to Dell et al*.* (2004), precipitation is a better proxy for ENSO than temperature in tropical regions because the latter variable does not change substantially in those regions, which diminishes its usefulness as a regressor. As a result, precipitation is employed in this study as a proxy for ENSO and is depicted in Fig. 1 below.^1^ To identify the directionality of the effect, we introduce a quadratic relationship between precipitation and common bean production. Thus, if only the linear part of the model is statistically significant, the relationship between precipitation and production goes in one direction: more (less) rainfall affect production. However, if the quadratic part of the model is also statistically significant, the relationship between precipitation and production depends on the level of precipitation: both excessive and insufficient rainfall have an effect on production.

Information on precipitation is obtained from the WorldClim data website, which is a database of high spatial resolution global weather and climate data computed from satellite information. These data have been downscaled from CRU-TS-4.03 by the Climatic Research Unit of the University of East Anglia, providing information on total precipitation in mm with a spatial resolution of 2.5 min per data point. We downloaded this information for the period 1991 to 2018 and computed monthly average precipitation levels for each municipality of the country. Using this information, three precipitation variables were computed. These variables differ in the geographical coverage considered to compute them. Thus, *precipitation1* contains the cumulative rainfall that occur in the entire Colombian territory during the whole year, *precipitation2* contains the cumulative rainfall that occur in the thirteen most important common bean production regions in Colombia during the whole year, and *precipitation3* contains the cumulative rainfall that occur in the thirteen most important common bean production regions in Colombia during the growing season, which typically occurs between April and September (Rios et al., 2017; Perez et al*.*, 2019). We chose thirteen regions because they represent nearly 90% of land area under common beans (DANE, 2014). In addition, we computed three precipitation variables to determine if rainfall distribution in the most important common bean production regions and during the growing season have a larger or smaller explanatory power for variations in common bean production than rainfall distribution in the whole country and for the whole year.

Several additional variables are introduced in the analysis to control for other phenomena that also affect common bean production apart from ENSO. According to FAO discussion in 2003, pre-2030 yield increases were going to be mainly driven by a sustained increase in fertilizer and pesticide use. Both fertilizer use per hectare and pesticide use per hectare increased in Colombia in the period under analysis, confirming FAO’s forecast that these products were going to have an increasing trend prior to 2030. We employ phosphorous/ha to capture fertilizer use for two reasons. On the one hand, phosphorus is a key input for common bean production, contributing substantially to the plant’s vegetative growth, pod formation and pod filling (Castro-Guerrero et al*.*, 2016). On the other hand, nitrogen use per hectare and potassium use per hectare have a similar trend than phosphorus per hectare. However, when all these variables are employed together in a regression model, a multicollinearity problem arises, which also affects the statistical significance of the ENSO indices since the latter variables are also correlated with input use. As a result, we run several regressions trying nitrogen, phosphorous, and potassium separately and selected the results associated with phosphorus/ha because this variable has the largest explanatory power among the three variables trialled. In addition, we include government consumption and private credit as Loayza et al*.* (2009) do. According to these authors, these two variables are important drivers behind the economic cycle and are expected to also affect the dynamics of particular agricultural products. Finally, we include the number of workers hired per ha of land and the total land area dedicated to agricultural production. These two variables have been found to highly affect agricultural production in Colombia (Ramirez-Villegas et al*.*, 2012; Feola et al*.*, 2015).

## Econometric approach

Loayza et al*.* (2009) propose the following model in differences to estimate the effects of ENSO on economic variables:

1 $${y}_{t}-{y}_{t-1}= {\beta }_{0}+{\beta }_{1}{EI}_{t}+controls+ {\varepsilon }_{t}$$

where ${EI}_{t}$ represents the index that captures the occurrence of ENSO, ${y}_{t}$ and ${y}_{t-1}$ represent the logarithm of any of the two endogenous variables of interest in this study at period $t$ and $t-1$, respectively, $controls$ represents all the additional variables introduced in the regression analysis that control for other phenomena different from ENSO variables, and ${\varepsilon }_{t}$ represents the residuals of the regression model. As the ENSO indices are highly correlated with precipitation, we estimate two separate models, one introducing ENSO indices and another only using precipitation variables. The model that only uses precipitation is the following:

2 $${y}_{t}-{y}_{t-1}= {\beta }_{0}+{\beta }_{1}({precip}_{t}-{precip}_{t-1})+ {\beta }_{2}({precip}_{t}^{2}-{precip}_{t-1}^{2})+controls+ {\varepsilon }_{t}$$

where ${\beta }_{1}$ captures the linear part of the relationship, ${\beta }_{2}$ the quadratic one, and ${precip}_{t}$ and ${precip}_{t-1}$ represent the logarithm of precipitation at period $t$ and $t-1$, respectively. According to Dell et al*.* (2014), the consistency of the estimated parameters in Eqs. (1) and (2) depends on the variables used to control for other factors that also affect common bean production apart from ENSO, which in turn determines the estimation technique to be employed. According to these authors, the inclusion of too many controls that are also correlated with ENSO indices create an overidentification problem, which generate a multicollinearity problem that may artificially reduce the statistical significance of the ENSO indices. In contrast, the inclusion of too few variables may create an omission variable bias, which generates that the residuals in Eqs. (1) and (2) become serially autocorrelated, affecting the consistency and unbiasedness of the estimated parameters. An omission variable bias also arises when the variables used as controls are not specific to common bean production but represent aggregate values for the whole agricultural sector. Table 1 shows the variables used in this study as controls and their definition.

Since FAO dataset does not contain information on input use for common bean production only, residuals in Eqs. (1) and (2) are expected to be serially correlated. Depending on the degree of the autocorrelation, several solutions are proposed (Greene, 2003, p. 250–282). As the residuals of Eqs. (1) and (2) exhibit a negative serial autocorrelation of degree one^2^ when estimated by OLS, we model residuals in the following way in order to deal with their serial autocorrelation:

3 $${\varepsilon }_{t}=\rho {\varepsilon }_{t-1}+{v}_{t}$$

where ${\varepsilon }_{t}$ and ${\varepsilon }_{t-1}$ represent the residuals of Eq. (1) or (2) at period $t$ and $t-1$, respectively, and ${v}_{t}$ is a white noise that follows a normal distribution with mean 0 and standard deviation ${\sigma }_{v}$. Under suitable manipulations of Eqs. (1) and (2), we estimate the following modified equations^3^:

4 $$\left({y}_{t}-{y}_{t-1}\right)-\rho \left({y}_{t-1}-{y}_{t-2}\right)= {\beta }_{1}\left({EI}_{t}-{\rho EI}_{t-1}\right)+{controls}_{t}-\rho {controls}_{t-1}+ {v}_{t}$$

5 $$\begin {aligned}\left({y}_{t}-{y}_{t-1}\right)-\rho \left({y}_{t-1}-{y}_{t-2}\right)= &\;{\beta }_{1}[\left({precip}_{t}-{precip}_{t-1}\right)-\rho \left({precip}_{t-1}-{precip}_{t-2}\right)\\&+{\beta }_{2}\left[\left({precip}_{t}^{2}-{precip}_{t-1}^{2}\right)-\rho \left({precip}_{t-1}^{2}-{precip}_{t-2}^{2}\right)\right]\\&+{controls}_{t}-\rho {controls}_{t-1}+ {v}_{t}\end {aligned}$$

Equations (4) and (5) are known as Prais-Winsten and Cochrane – Orcutt error correction models (Davidson & MacKinnon, 1993, p. 343–351). They aim at removing the autocorrelation of degree 1 in the residuals, which is addressed by multiplying the lag of Eqs. (1) and (2) by $\rho$ and subtracting the latter multiplication from the original equations, which result in Eqs. (4) and (5). This computation is performed with the aim of obtaining the expression ${\varepsilon }_{t}-\rho {\varepsilon }_{t-1}={v}_{t}$, which results to be a white noise that is normally distributed with mean 0 and standard deviation ${\sigma }_{v}$. In this formulation, $\rho$ is a parameter that cannot be estimated using traditional OLS formulas. Cochrane and Orcutt (1949) propose a methodology to compute $\rho$ optimally. This algorithm makes part of the *prais* function in STATA. The prais function allows determining several search approaches to determine the optimal value of $\rho$. We have set this algorithm to search for a value of $\rho$ that minimizes the sum of the squared errors (SSE) with the aim of obtaining the most efficient estimate for this parameter. Based on the estimated values for $\rho$, we have also computed a Durbin-Watson test to the corrected residuals to determine if the autocorrelation problem is reduced by the application of this estimation methodology. Durbin-Watson tests applied to both the original residuals and the corrected ones are shown in Table 4.

Except for the ENSO indices, all variables used in this study are introduced in the regression analysis in differences because they result to be non-stationary. Table 3 shows Dickey-Fuller tests applied to both endogenous variables and regressors.^4^ The null hypothesis for a Dickey-Fuller test is whether the time series under analysis is non-stationary. Hence, lacking to reject the null hypothesis implies that there is empirical evidence that the time series under analysis is non-stationary (Greene, 2003). As can be seen in Table 3, all variables except yield/ha and the precipitation variables are non-stationary, but their first difference result to be stationary. Davidson and MacKinnon (1993) show that spurious estimations are obtained from regressions that run stationary against non-stationary variables. This is corrected by running non-stationary variables in differences when the latter transformations are stationary. In addition, it is customary to run all variables in differences when some of them must be run in differences in order to facilitate the interpretation of the results. As the difference of a variable in logarithms from one period to the next one is approximately equal to the variable’s growth rate, estimated parameters can be interpreted as indicating how much the growth rate of an endogenous variable changes with a marginal percentage change in a regressor.

**Table 3:** Dickey-Fuller Tests for Non-Stationarity

Variables^ab^	In levels	In differences
yield/ha	-3.03**	-7.62***
Income	-2.54	-6.49***
phosphorus/ha	-1.71	-4.06***
fungicide/ha	-2.53	-7.80***
pesticide/ha	-1.49	-4.57***
gov. consumption	-1.41	-4.25***
workers/ha	-2.02	-3.67***
private credit	-0.05	-3.58***
agricultural land	-0.45	-4.48***
precipitation1	-3.45***	-6.64***
precipitation2	-3.51***	-7.30***
precipitation3	-4.34***	-8.73***

^a^ All variables are tested in logarithms

^b^ A trend and several lags were introduced in the Dickey-Fuller Tests performed; Any of these variables resulted statistically significant

*** Statistically significant at 1%; ** Statistically significant at 5%; * Statistically significant at 10%

## Results

Table 4 presents the econometric estimations for Eq. (3) and (4). As we employ two different ENSO indices and three different precipitation variables, there are five regression results per endogenous variable considered. The results in the table indicate that yield/ha is mainly affected by El Niño. The growth rate of common beans’ yield/ha was 9% smaller during El Niño events in the period 1991 to 2018. This implies that both the generalised increment in temperatures and the generalised reduction in precipitation that occur in Colombia during El Niño phase reduce common beans’ yields. This result does not help elucidate though whether excessive rainfall (floods) or deficient rainfall (droughts) is the main driver behind yield reduction since the ENSO indices do not perfectly explain the extreme instances of precipitation. When cumulative precipitation is employed as a regressor, the results indicate that the quadratic term associated with cumulative precipitation is statistically significant, but the linear term is not. This implies that deviations in the growth rate of total precipitation with respect to its long-run growth rate reduce common beans’ yields/ha in Colombia, regardless of the measure of cumulative precipitation used for the estimations. These deviations are associated with the generalised increment in cumulative precipitation that occurs in Colombia during La Niña and the generalised reduction in cumulative precipitation that occurs in Colombia during El Niño. Hence, a positive deviation in cumulative precipitation can be understood as an excess in rainfall due to La Niña and a negative deviation in cumulative precipitation can be understood as a deficit in rainfall due to El Niño. The linear term would have added a lower bound for the deviation to start affecting yield/ha. Since the linear term is not statistically significant for the relationship between precipitation and yield/ha and the average growth rate for precipitation^5^ is around 0, temporary deviations in the growth rate of cumulative precipitation from 0^6^ reduce common beans’ yield/ha in Colombia. This result is stronger when only the most important common bean production regions are considered and the timeframe for precipitation is restricted to the growing season, which is observed with the increasing significance of the variables of precipitation introduced in the study, where the last one (*precipitation3*) captures the cumulative precipitation that occurred in the thirteen most important common bean production regions during the growing season.

**Table 4:** Regression Results using a Prais-Winsten Error Correction Model for the period 1991–2018

Regressors^a^	yield/ha^b^ (kg/year)					income^b^ (LCU^d^, 2016 = 100)				
index_ENSO^c^	-0.06**					0.08				
index_La Niña^c^		-0.03					0.11			
index_EL Niño^c^		-0.09**					0.04			
precipitation1			0.003					0.09		
precipitation1^2			-0.81**					1.60		
precipitation2				0.05					0.06	
precipitation2^2				-0.54**					1.63**	
precipitation3					-0.03					0.13
precipitation3^2					-0.25***					0.81***
phosphorus/ha	-0.002	-0.01	-0.01	-0.01*	-0.01	-0.07	-0.07	-0.06	-0.05	-0.05
fungicide/ha	0.003	0.01	0.09	0.08	0.09					
pesticide/ha	0.03	0.03	0.005	-0.001	-0.003					
gov. consumption						-0.59	-0.44	-0.54	-0.55	-0.45
workers/ha (lag)						-20.03**	-20.36**	-22.26**	-22.59**	-22.65***
private credit (lag)						1.07***	1.10***	0.98**	0.91**	0.93**
agricultural land						-3.39***	-3.07**	-3.57**	-3.30**	-3.28**
R^2^	0.21	0.28	0.17	0.18	0.15	0.56	0.56	0.56	0.58	0.62
DW Test (original)	2.67	2.64	2.73	2.71	2.63	2.89	2.90	2.80	2.83	2.96
DW Test (transformed)	2.22	2.19	2.19	2.27	2.17	2.12	2.11	1.96	1.98	2.04
Rho	-0.45	-0.44	-0.50	-0.51	-0.43	-0.70	-0.70	-0.68	-0.68	-0.73

^a^ Regressors used result from taking logarithms to the original variables and then taking the difference of the variables in logarithms; resulting variables are all stationary

^b^ Endogenous variables used result from taking logarithms to the original variables and then taking the difference of the variables in logarithms; resulting variables are all stationary

^c^ Indices are introduced as defined in Table 1.

^d^ *LCU* Local Currency Units

*** Statistically significant at 1%; ** Statistically significant at 5%; * Statistically significant at 10%

In turn, farmers’ income from common bean production is not affected by any of the ENSO indices computed. This result does not immediately imply that ENSO does not affect farmers’ income from common bean production. What it indicates is that the total income obtained by common bean growers during the extreme phases of ENSO does not differ from the income obtained during the neutral phase. Hence, there seems to exist other phenomena during the neutral phase that affect farmers’ income from common bean production in such as way as to make it similar to the income received by this farming community during the extreme phases of ENSO. These other phenomena may also be associated with climatic changes such as changes in precipitation, which are not captured by a set of indices that only are capable of capturing the occurrence of an extreme event at one particular point in time. In that sense, using cumulative precipitation as a regressor is more appropriate since this variable is capable of capturing dynamic changes in the weather that are not captured by the type of ENSO indices computed in this study. When cumulative precipitation is employed, the results indicate that the quadratic term is statistically significant, but the linear term is not, as occurs to yield/ha. However, the coefficient associated with the quadratic term is positive in this case, which is contrary to what happens to yield/ha. Hence, temporary deviations in the growth rate of cumulative precipitation from its long-run rate increase farmers’ income from common bean production rather than decrease it. This result implies that farmers receive less income from common bean production during those years in which the levels of rainfall are stable from year to year than on those years in which the levels of rainfall are vey unstable from year to year. In other words, slight movements towards more or less rainfall in a particular year relative to the average level generate an increment in the income obtained by farmers from common bean production. This result helps explain why the ENSO indices are not statistically significant to explain the variability of income from common bean production. Farmers’ income from common bean production seems not to be affected by the extreme phases of ENSO because it is highly affected by the initial phases of the change in the weather, which necessarily occurs during the neutral phase of ENSO. As a result, farmers’ income from common bean production during the neutral phase appears to be as affected by changes in cumulative precipitation as farmers’ income from common bean production during the extreme phases of ENSO.

All these results suggest that temporary deviations in the growth rate of cumulative precipitation from its long-run rate reduce yield/ha and increase farmers’ income from common bean production. This in turn implies that Colombian farmers transfer the whole effect of ENSO on common bean production to consumers. In fact, a positive relationship between deviations in cumulative precipitation and farmers’ income indicates that reductions in yield/ha are more than compensated by increases in prices/kg of common beans, inducing an extra transfer of consumers’ surplus to farmers as a compensation for the ENSO-related weather shocks. In addition, this result becomes stronger when only the most important common bean production areas are considered, and precipitation is restricted to the growing season.

## Discussion

The next few decades predict significant disruption for tropical zones due to increased ENSO effects (Bruinsma, 2003). The common bean can be regarded as a resilient crop that offers multiple nutritional benefits for low income and subsistence households but that is affected by extreme weather events such an ENSO. This paper aims to understand the economic disruption caused by ENSO to common bean growers who are located in a tropical zone that is on the brink of a transition towards worsened climatic factors. This increasing severity may have implications for economic development and food security, as well as for farmers’ capacity for self-sufficiency. We find evidence of a negative impact of ENSO on common beans’ yields in the period 1991 to 2018, with El Niño having the largest effect on yields. We also find evidence that yields were negatively affected by excessive cumulative precipitation and by deficient cumulative precipitation in the period under analysis, which implies that common beans’ yields are reduced when annual cumulative precipitation is more than normal and when is also less than normal. In other words, common bean yields grow optimally when climatic conditions are optimal, which requires that the plants neither receive too much rainfall nor too few.

In turn, we find evidence that the level of precipitation is a very important determinant of farmers’ income from common bean production. Our results show that farmers’ income from common bean production changes positively when the climatic conditions become wetter or drier than normal, and that slight changes in cumulative precipitation seem to have a similar effect on income than large changes in cumulative precipitation induced by the extreme phases of ENSO. Consequently, this paper shows that ENSO has had a substantial impact on common bean production in Colombia prior to 2030, where common beans’ yields and farmers’ income from common bean production have been impacted with the climatic phenomenon.

This paper also contributes to the “new climate-economy literature” (Dell et al*.*, 2014) in two respects. On the one hand, it provides the first application of time series to determine the effect of ENSO on common bean production in Colombia. Current applications are addressed to investigate the effect of ENSO on agricultural production and inflation in the region ((MinAgricultura, 2012; Melo-León et al*.*, 2017; Smith & Ubilava, 2017; Abril-Salcedo et al*.*, 2018). Some papers have investigated the effect of ENSO on particular crops, such as coffee (Bastianin et al*.*, 2018), citrus (Gonzalez-Orozco et al*.*, 2020), oil palm tree (Cadena et al*.*, 2006), avocado (Rodriguez-Gil et al*.*, 2020), and tilapia (Blanco et al*.*, 2007). This paper adds to this literature by investigating the effect of ENSO on common beans, focusing on yields/ha and farmers’ income from common bean production. On the other hand, this paper advances in the investigation of the relationship between precipitation and common bean production in Colombia. Previous studies have not attempted to determine if there exists a quadratic relationship between agricultural production and precipitation, even though it is suggested in the new climate-economy literature. This paper is the first one in investigating the relationship between precipitation and agricultural production among the existing papers for the region.

The Colombian government could employ the results of this paper to design public policy. In particular, it can design countercyclical policy aimed at reducing price volatility in the Colombian market of common beans. If common beans are truly seen as a mechanism to provide highly nutritious staple foods to low-income consumers in Colombia, the government may, for instance, open the economy to the international market in order to regulate the price in the internal market; a solution proposed by FAO (Bruinsma, 2003) as a measure to reduce price volatility and increase food security in the economies affected by climatic phenomena such as ENSO.

An important consequence of the results obtained in this paper is that the focus of bean breeders and companies that develop new phenotypes of common beans should be on developing genotypes that are both resistant to droughts and biotics stresses generated by excessive rainfall. Most of the efforts carried out by these institutions have been on developing phenotypes that are drought- and heat resistant since these two phenomena are expected to be the most prevalent after 2050 in Colombia (Ramirez-Villegas et al*.*, 2012; Beebe et al., 2011; Porch et al., 2013). However, the results of this paper indicate that the growth rate of common beans’ yield/ha responds negatively to both excessive and insufficient rainfall. As an excess in rainfall is correlated with an increased prevalence of plants’ pests and diseases and a deficit in rainfall is correlated with droughts (Rosenzweig et al*.*, 2001; Ramirez-Villegas et al*.*, 2012; Feola et al*.*, 2015), new common bean genotypes should be developed to be resistant to phenomena that arise in both extreme phases of ENSO: droughts, heat, and biotic stresses, such as pests and diseases.

## Conclusions

The common bean is an important staple food in Colombia. Not only common beans are an excellent source of nutrients for low-income consumers, they also are an excellent tool to reduce GHG emissions from agricultural production in the country. However, climate change and its extreme climatic events, such as El Niño Southern Oscillation (ENSO), threaten the use of common beans as a tool to increase food security of the poor and reduce emissions from the agricultural sector. In order for the Colombian government to be able to design public policy to counteract the negative effects of ENSO on food security, risk assessments of the effect of ENSO on common beans are required. This paper performs the first quantitative evaluation of the effects of ENSO on common bean production in Colombia. By utilising information on common beans’ yield/ha and farmers’ income from common bean production in Colombia for the period 1991 to 2018 from FAO dataset, this paper carries out the first econometric estimation of the effects of ENSO on common bean production in Colombia using a time series approach.

The results of the paper show that El Niño phase is the most important driver behind the growth rate of common beans’ yield/ha in Colombia in the period under analysis. The growth rate of common beans’ yield/ha was 9% smaller during El Niño events in the period 1991 to 2018. The results also show that ENSO-induced changes in cumulative precipitation also generate a reduction in common beans’ yields, regardless of the sign of the change. In other words, common beans’ yield/ha is negatively affected by excessive and insufficient cumulative precipitation, which implies that yields and precipitation have a quadratic relationship. In turn, the results also show that precipitation and farmers’ income from common bean production have a quadratic relationship. In other words, deviations in the growth rate of precipitation from its long run rate generate an increase in the income obtained by farmers from common bean production, regardless of the sign of the deviation. Thus, both excessive and insufficient rainfall induce a reduction in yield/ha and an increase in income from common bean production, which implies that reductions in yields given by ENSO are overcompensated by price spikes in the kg of common beans. The latter also implies that ENSO events generate a transfer of consumers’ surplus to common beans’ growers, which worsen consumers’ purchasing power and food security during the climatic event.

These results imply that ENSO has a larger negative impact on consumers than on growers in Colombia. Consequently, Colombian policy makers could introduce policy instruments that help stabilise the price of common beans during both extreme phases of ENSO, placing particular attention to EL Niño phase since this phase has the largest negative impact on yields. For instance, the Colombian government could facilitate the importation of common beans from markets such as the US, Canada, or Argentina, in order to increase competition in the Colombian market and incentivise a reduction in the price of common beans during EL Niño phase. This facilitation needs to be gradual and measured in order not to affect farmers’ incomes during the worst part of EL Niño phase, when farmers are highly affected by rainfall shortages. The Colombian government could also design long-run programmes that incentivise the uptake of climate-adapted common bean seeds. These seeds are being developed by centres such as *Centro International de Agricultura Tropical* (CIAT) or *Corporación Colombiana de Investigación Agropecuaria* (AGROSAVIA), but they have had a very low uptake in rural areas of Colombia (Botero et al*.*, 2021). A policy option would be to provide financial aid to farmers so they can switch smoothly from traditional varieties to the climate-adapted ones developed by these centres.

The results of this study also indicate that future research should also consider the consequences of very wet conditions on yields. As excessive precipitation appears to have a negative effect on the yields of common beans in Colombia, future research should also attempt to determine which gene traits are utilised by common beans to adapt to very wet conditions, if any. CIAT and AGROSAVIA could also attempt to develop varieties that grow well in very wet conditions. Socioeconomic research could be focused on determining the strategies pursued by common bean farmers to adapt to ENSO events. The identification of these strategies may inform the type of strategy that the Colombian government could pursue to increase the uptake of climate-adapted common bean varieties by Colombian farmers.

## Footnotes

1 We computed an ENSO index based on the distribution of precipitation. The correlation coefficient between the resulting indices and the ENSO indices based on the monthly SOI is 0.72. This result is explained by the fact that the ENSO indices based on the distribution of precipitation only capture the extreme effects of ENSO on precipitation, which generally occur within a year, whereas the ENSO indices based on the monthly SOI capture variations on sea surface temperature, which is not immediately or directly reflected in changes in the distribution of rainfall in Latin America.

2 We computed Durbin-Watson tests to determine whether autocorrelation was a problem of the OLS regressions. These tests are provided in Table 4.

3 We use *controls* as a shorthand writing for all other variables included in the model. As all variables are introduced in differences (see below), ${controls}_{t}$ refers to the difference of the logarithms of the original variables at period $t$ (e.g., the control variable for fungicide is: ${control}_{t}={fungicide}_{t}-{fungicide}_{t-1}$, where *fungicide* is expressed in logarithms).

4 The ENSO indices are all stationary because they only assume two values: 1 and 0.

5 See Table 2.

6 The long-run growth rate of precipitation.
